# MRI/Transrectal Ultrasound Fusion-Guided Targeted Biopsy and Transrectal Ultrasound-Guided Systematic Biopsy for Diagnosis of Prostate Cancer: A Systematic Review and Meta-analysis

**DOI:** 10.3389/fonc.2022.880336

**Published:** 2022-05-23

**Authors:** Jianfeng Xie, Chunchun Jin, Mengmeng Liu, Kun Sun, Zhanqiang Jin, Zhimin Ding, Xuehao Gong

**Affiliations:** ^1^Department of Ultrasound, Southern University of Science and Technology Hospital, Shenzhen, China; ^2^Department of Ultrasound, The First Affiliated Hospital of Shenzhen University, Shenzhen Second People’s Hospital, Shenzhen, China; ^3^Department of Ultrasound, First Affiliated Hospital of Southern University of Science and Technology, Second Clinical College of Jinan University, Shenzhen Medical Ultrasound Engineering Center, Shenzhen People’s Hospital, Shenzhen, China

**Keywords:** magnetic resonance imaging, transrectal ultrasound, prostate cancer, targeted biopsy, meta-analysis

## Abstract

**Purpose:**

For men suspected of having prostate cancer (PCa), the transrectal ultrasound (TRUS)-guided systematic biopsy (SB) was performed. MRI/TRUS fusion guided-targeted biopsy (MRI-TB) could enhance PCa detection, allowing sampling of sites at higher risk which were not obvious with TRUS alone. The aim of this systematic review and meta-analysis was to compare the detection rates of prostate cancer by MRI-TB or MRI-TB plus SB versus SB, mainly for diagnosis of high-risk PCa.

**Methods:**

A literature Search was performed on PubMed, Cochrane Library, and Embase databases. We searched from inception of the databases up to January 2021.

**Results:**

A total of 5831 patients from 26 studies were included in the present meta-analysis. Compared to traditional TRUS-guided biopsy, MRI-TB had a significantly higher detection rate of clinically significant PCa (RR=1.27; 95%CI 1.15-1.40; p<0.001) and high-risk PCa (RR=1.41; 95% CI 1.22-1.64; p<0.001), while the detection rate of clinically insignificant PCa was lower (RR=0.65; 95%CI 0.55-0.77; p<0.001). MRI-TB and SB did not significantly differ in the detection of overall prostate cancer (RR=1.04; 95%CI 0.95-1.12; p=0.41). Compared with SB alone, we found that MRI-TB plus SB diagnosed more cases of overall, clinically significant and high-risk PCa (p<0.001).

**Conclusion:**

Compared with systematic protocols, MRI-TB detects more clinically significant and high-risk PCa cases, and fewer clinically insignificant PCa cases. MRI-TB combined with SB enhances PCa detection in contrast with either alone but did not reduce the diagnosis rate of clinically insignificant PCa.

**Systematic Review Registration:**

https://www.crd.york.ac.uk/prospero/#searchadvanced, CRD42021218475.

## 1 Introduction

Prostate cancer (PCa) is the most common type of cancer in men (1). Conventional methods for diagnosis of prostate cancer include prostate-specific antigen (PSA) blood examinations and digital rectal examination (DRE). Diagnosis using these two methods is confirmed through transrectal ultrasound(TRUS)-guided systematic prostate biopsy (SB) (2). However, low sensitivity and specificity of conventional TRUS-guided biopsy limits its application (3). In addition, TRUS-guided biopsy does not detect approximately 20% of significant PCa (csPCa) cases (4). Moreover, TRUS-guided biopsy over detects clinically insignificant PCa (cisPCa), increasing overtreatment and associated side events like erectile dysfunction and urinary incontinence (5).

Multiple methods have been developed based on MpMRI (multiparametric magnetic resonance imaging) to obtaining prostate biopsy cores. Currently, MRI-guided in-bore biopsies (within the MRI machine) are available, but they are costly and time consuming, particularly given the requirement of non-ferromagnetic biopsy instruments in the MRI setting (6). Cognitive fusion is a process where the examining specialist assesses MRI images before ultrasound (US)-guided assessment, reduces costs as well as the spatial correlation capacity (7). Therefore, platforms which allow instant mpMRI and US image fusion biopsy have several advantages, such as implementation outside the MRI setting, good spatial correlation and are not costly (8). MRI-TRUS fusion guided-targeted biopsy (MRI-TB) combines mpMRI’s high accuracy in lesion detection and characterization and cost-efficiency with the simple-to use TRUS-guided platforms for urologists (9, 10). A previous study reports that targeted biopsy using mpMRI-TRUS fusion enhances PCa detection, allowing sampling of sites at higher risk which could not be detected with TRUS alone (11). In addition, it reduces over detection of insignificant tumors, thus avoiding unwarranted radical treatment (11).

Several meta-analyses have been published on diagnostic approaches of prostate cancer (12, 13). However, they focused on evaluating diagnosis of clinically significant and insignificant PCa, excluding high-risk PCa and did not explore benefits of MRI-TB in combination with SB. High-risk prostate cancer is an aggressive disease characterized by relapse after definitive treatment (14). Therefore, the aim of this systematic review was to compare the detection rates of prostate cancer by MRI-TB or MRI-TB plus SB versus SB in men with high serum PSA levels and/or abnormal digital rectal examination, mainly for diagnosis of high-risk PCa.

## 2 Methods

### 2.1 Literature Search

PRISMA (Preferred Reporting Items for Systematic Reviews and Meta-Analyses) guidelines (15) were followed in this systematic review and meta-analysis. The study was registered with the international prospective register of systematic reviews (PROSPERO, ID CRD42021218475). Related studies were searched in PubMed, Cochrane Library, and Embase databases without any restriction on publication language. The search key words included prostate cancer (prostate neoplasm), MRI, ultrasonography, image-guided biopsy. Related studies were retrieved from the inception of the database to January 2021.

### 2.2 Inclusion and Exclusion Criteria

Paired retrospective and prospective articles were included in this study. Patients with suspected PCa showed increased serum PSA (prostate-specific antigen) contents, uncertain results from digital rectal assessment, and were biopsy naïve or reported previous negative biopsy. Studies included in this review explored MRI-TB and transrectal SB, to determine the effectiveness of MRI-TB in detection of PCa compared with traditional SB (SB relative to MRI-TB, and/or MRI-TB+SB relative to SB). Moreover, only studies with SB standard of 12 or close to 12 (12 ± 2) cores and those guided by the MRI/TRUS fusion prostate model or TRUS were included. Studies not published in English, studies involving proven PCa cases on active surveillance, studies involving transperineal biopsy, studies not comparing MRI-TB vs SB, studies that did not report outcomes, non-primary studies, literature reviews, and meta- analyses were excluded from this study. No standard definition of clinically significant, and clinically insignificant cancers exists, therefore, definitions of such cases in respective articles were allowed. In the absence of a clear definition, where applicable Gleason grade ≥3+4 cancer was regarded clinically significant, whereas Gleason grade 3 + 3 was regarded clinically insignificant (16). Similarly, it is also hard to define the high risk PCa.The American Urological Association (AUA) defines “high-risk” as a clinical T stage ≥cT2c, a Gleason score≥8, or a PSA >20 ng/mL. The National Comprehensive Cancer Network (NCCN) defines “high-risk” as T3a, Gleason ≥8, or PSA ≥20, and “very high risk” as T3b or T4 disease.High risk in theThe Radiation Therapy Oncology Group (RTOG) classification includes 1) Gleason ≥8, or 2) Gleason =7 plus either ≥cT3 or node-positive. Included articles that met any of the above definitions could be considered high-risk prostate cancer (17).

### 2.3 Study Selection and Extraction of Data

Data retrieval was performed by 2 independent reviewers and discrepancies between them were solved through discussions or consulting a third reviewer. Titles and abstracts were examined for relevance before full-text reviews of articles. Information on the article, participant, MRI, as well as biopsy features were recorded in a standard form as follows: 1) article: origin (authors, publication year), definition of csPCa, definition of high risk PCa; 2) study participant: clinical setting (prior negative biopsy or biopsy naïve), number of participants (overall), MRI sequence for defining target, age, mean prostate volume, and mean PSA; 3) MRI: magnet strength, MRI sequences, and number of patients with positive MRI scan; 4) biopsy: core numbers and lesion numbers for MRI-TB/SB, previous biopsy status, rates of detection of csPCa, overall PCa, cisPCa, as well as high risk PCa.

### 2.4 Quality Assessment of Included Studies

Two reviewers independently assessed the risk of bias and applicability concern in a single study using a revised tool for Quality Assessment of Diagnostic Accuracy Studies (QUADAS-2) checklist. RCTs were assessed using Cochrane risk of bias 2.0 tool to evaluate the risk of bias.

### 2.5 Statistical Analysis

All statistical analyses were performed with Stata V.15 software. Relative risk (RR) and 95% CI were implemented to characterize the dichotomous variables comprising overall PCa, clinically insignificant PCa, clinically significant PCa, and high risk PCa. Q and I² statistics were employed to assess study heterogeneity. The random-effect model was implemented where P<0.1 or I²>50%, whereas fixed-effect model was used when this threshold was not met. Sensitivity analyses were performed by eliminating one article consecutively to validate the stability of final results. Subgroup analyses were performed based on available information. Egger’s and Begg’s tests were used to determine publication bias. p<0.05 represented statistical significance, and all tests were two-sided.Subgroup assessments were carried out based on prior biopsy status due to potential heterogeneity. Studies were then divided into 3 sub-groups including a previous prostate cancer biopsy that was negative, biopsy naïve, and mixed prostate cancer biopsy.

## 3 Results

### 3.1 Summary of Studies

**Figure 1:**
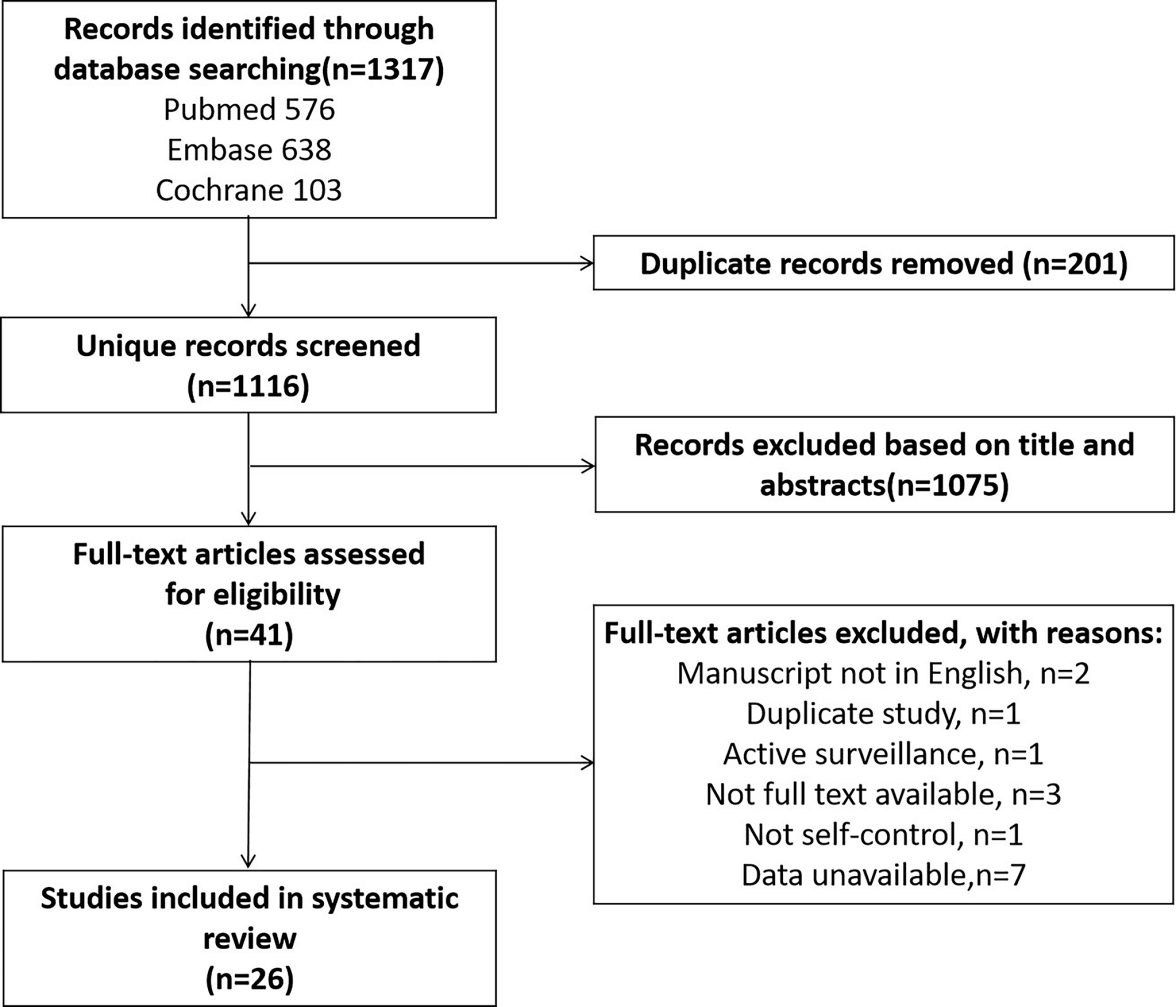
Flowchart of the search strategy.

A total of 1317 articles were retrieved. After elimination of duplicates, and review of titles and abstracts, and full-text review, a total of 26 articles including 5831 PCa cases were included in this study (**Figure 1**).

### 3.2 Characteristics of Enrolled Studies

**Figure 2:**
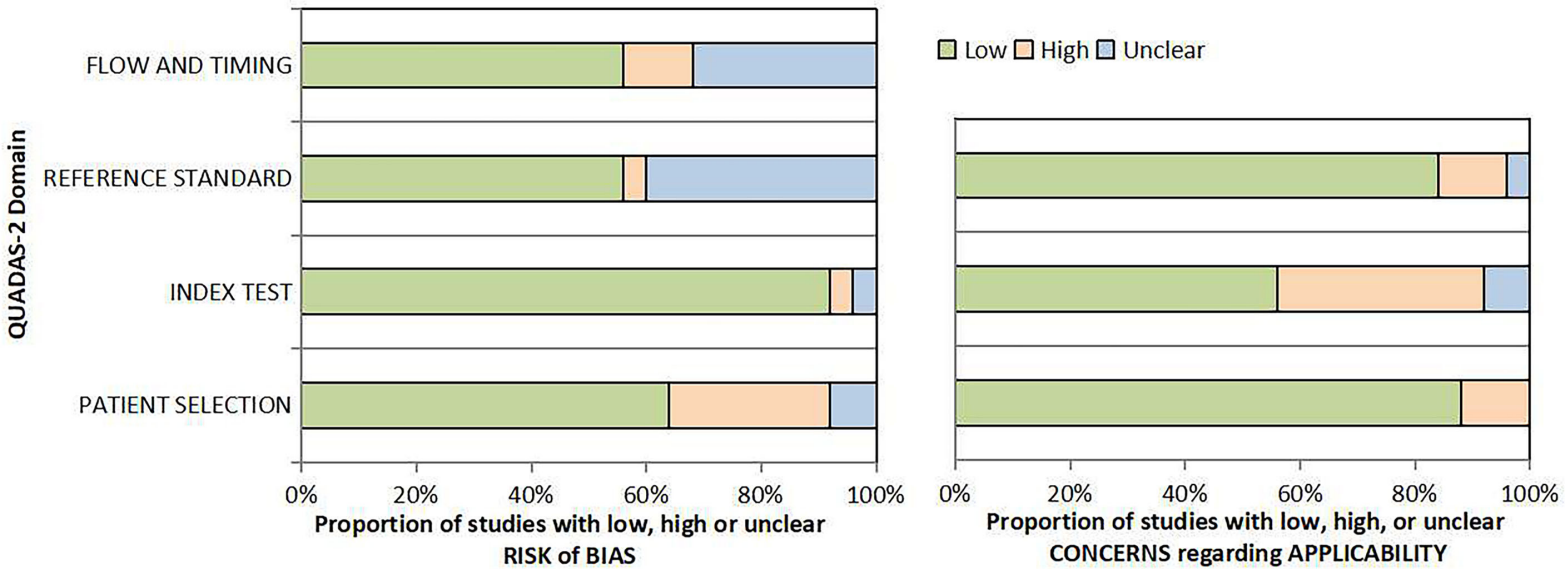
Quality assessment according to QUADAS-2 of the included studies.

Out of the 26 enrolled studies, 25 used paired designs and 1 was a RCT. MRI-TB was used in all MRI navigation methods. Six studies included biopsy naïve patients, whereas six studies enrolled participants with prior negative biopsy results. The other studies used mixed biopsy, however, one study did not specify the biopsy type. 23 of the included studies provided clinically significant PCa’s definition and reported its detection rate. Some articles defined clinically significant PCa based on Gleason score ≥7 or >2 positive cores. Some studies used Gleason score with other criteria based on core information (maximum cancer core length ≥4 mm). The sample size in each article ranged between 33 and 1042. Patient age ranged from 59 to 70 years. 1.5-T or 3.0-T scanner was used for mp-MRI examination in all included studies. Each patient presented with at least one disputable lesion as shown by MRI results, and each lesion was obtained from at least one targeted core. Systematic biopsies from the same session were carried out using a median of 10 - 13 cores through the transrectal route. Main features of included studies and PCa cases are presented in **Table 1**. The quality assessment of these enrolled studies is shown in **Tables 2**, **3** and **Figure 2**.

**Table 1:** Clinical characteristics of include studies.

Author	Year	Population	No.of patients	Mean age (yr)	Mean PSA (ng/ml)	Mean prostate volume (cc)	Positive MRI	Field of strength (Tesla)	MRI sequences	Endorectal coil	Target approach (cores per target)	Comparator (cores)	Definition of clinically significant PCa
Jelid et al. (18)	2017	Biopsy naïve + prior negative biopsy	130	62.9	9.5	45.9	130	3	T2, DWI, DCE	Yes	Fusion-TBx (2–3)	TRUS-Bx (16)	GS>7 or MCCL≥5 mm for GS 6
Labra et al. (19)	2020	Biopsy naïve + prior negative biopsy	122	63	5.8	NR	122	3	T2, DWI, DCE, ADC	No	Fusion-TBx (6)	TRUS-Bx (13)	GS ≥ 3 + 4
Rastinehad et al. (20)	2014	Biopsy naïve + prior negative biopsy	105	65.8	7.52	NR	105	3	T2,ADC, DCE	NR	Fusion-TBx (NR)	TRUS-Bx (12)	GS ≥ 3 + 4 or GS >6 or 6 with 50% involvement of PCa per core
Junker et al. (21)	2015	prior negative biopsy	50	63.7	7.6	49.2	50	3	T2, DWI, DCE	No	Fusion-TBx (3-5)	TRUS-Bx (10)	NR
Baco et al. (22)	2016	Biopsy naïve	175	65	7.3	42	86	1.5	T2,ADC, DCE	NR	Fusion-TBx (2)	TRUS-Bx (12)	MCCL ≥ 5 mm for GS 6 or any MCCL for GS≥7 disease
Filson et al. (23)	2016	Biopsy naïve + prior negative biopsy+prior positive biopsy	1042	65	6	48.6	825	3	T2, DWI, DCE, ADC	NO	Fusion-TBx (NR)	TRUS-Bx (12)	Gleason score ≥ 7
Mariotti et al. (24)	2017	Biopsy naïve + prior negative biopsy	100	62.5	5.3	NR	100	3	T2, DWI, DCE, ADC	NO	Fusion-TBx (NR)	TRUS-Bx (14)	Gleason score ≥ 3 + 4
Mariotti et al. (25)	2016	Biopsy naïve + prior negative biopsy	389	62.8/62.7	8.0/6.4	40/64	389	3	T2, DWI, DCE	NO	Fusion-TBx (2-3)	TRUS-Bx (12)	GS 3 + 4 with 50% or more of any core positive for cancer or 33% or more of standard biopsy cores positive for cancer
Kongnyuy et al. (26)	2017	Biopsy naïve + prior negative biopsy+prior positive biopsy	195	60.3	7.8	49	195	3	T2, DWI, DCE	Yes	Fusion-TBx (2)	TRUS-Bx (12)	Gleason score≥3+4
Siddiqui et al. (27)	2015	Biopsy naïve + prior negative biopsy	1003	62	10	59.5	1003	3	T2, DWI, DCE	Yes	Fusion-TBx (5)	TRUS-Bx (12)	GS 3 + 4 with 50% or moreof any core positive for cancer or 33%
Author	Year	Population	No.of patients	Mean age (yr)	Mean PSA (ng/ml)	Mean prostate volume (cc)	Positive MRI	Field of strength (Tesla)	MRI sequences	Endorectal coil	Target approach (cores per target)	Comparator (cores)	Definition of clinically significant PCa
Brock et al. (28)	2015	prior negative biopsy	168	64	9.2	55.4	168	3	T2	No	Fusion-TBx (2)	TRUS-Bx (12)	GS>6 and/or 6 with 50% involvement of PCa per core
Mozer et al. (29)	2015	Biopsy naïve	152	63	6	44	152	1.5	T2, DWI, DCE	No	Fusion-TBx (2-3)	TRUS-Bx (12)	at least one core with a GS of 3 + 4 or 6 with a MCCL ≥4 mm.
Delongchamps et al. (30)	2016	Biopsy naïve	108	65	7.2	46	108	1.5/3.0	T2, DWI, DCE	Yes	Fusion-TBx (3)	TRUS-Bx (10-12)	MCLL≥5 mm for GS 6 or any GS≥7 disease
Kaushal et al. (31)	2019	prior negative biopsy	131	63.5	9.75	54.2	131	3	T2, DWI, DCE	NR	Fusion-TBx (1-2)	TRUS-Bx (12)	Gleason score ≥4+3
Fujii et al. (32)	2019	Biopsy naïve	131	70	6.51	40.3	131	1.5/3.0	T2, DWI, DCE, ADC	NR	Fusion-TBx (2-5)	TRUS-Bx (10)	a single biopsy core showing disease of GS ≥4 + 3 and/or MCCL ≥ 6mm
Salami et al. (33)	2015	prior negative biopsy	140	66.3/65	9.7/7.6	50/54.5	140	3	T2, DWI, DCE, ADC	Yes	Fusion-TBx (NR)	TRUS-Bx (12)	GS ≥ 7, or GS 6 with > 2 cores positive and/or >50% of any core involved with cancer or GS 6 with a lesion volume > 0.2 cm3
Vourganti et al. (34)	2012	prior negative biopsy	195	62	9.13	56	195	3	T2, DWI, DCE, ADC	Yes	Fusion-TBx (NR)	TRUS-Bx (12)	Gleason score ≥7
Yarlagadda et al. (35)	2018	Biopsy naïve	69	64.33	7.71	54.26	69	3	T2, DWI, DCE	NR	Fusion-TBx (1-9)	TRUS-Bx (12)	NR
Zhu et al. (36)	2018	NR	998	59.12	4-10/10.1-20	58.52	998	3	T2, DWI, DCE, ADC	Yes	Fusion-TBx (9)	TRUS-Bx (12)	NR
Gorski et al. (37)	2015	Biopsy naïve	232	64	6.65	40	232	1.5	NR	NR	Fusion-TBx (2-3)	TRUS-Bx (12)	at least 1 core with a GS of 7 (3 + 4) or 6 with MCCL ≥4mm
Author	Year	Population	No.of patients	Mean age (yr)	Mean PSA (ng/ml)	Mean prostate volume (cc)	Positive MRI	Field of strength (Tesla)	MRI sequences	Endorectal coil	Target approach (cores per target)	Comparator (cores)	Definition of clinically significant PCa
Fiard et al. (8)	2013	Biopsy naïve + prior negative biopsy	30	64	6.3	46	20	3	T2, DWI, DCE, ADC	NR	Fusion-TBx (2)	TRUS-Bx (12)	Gleason score ≥ 4 + 3 or total cancer length on biopsy ≥10 mm
Sonn et al. (38)	2013	prior negative biopsy	105	65	7.5	58	105	3	NR	No	Fusion-TBx (1-9)	TRUS-Bx (12)	Gleason ≥ 3 + 4 or Gleason 6 with MCLL≥4 mm
Wysock et al. (39)	2013	Biopsy naïve + prior negative biopsy	125	65	5.1	46	125	3	T2, DWI, DCE	No	Fusion-TBx (2)	TRUS-Bx (12)	Gleason score ≥7
Ukimura et al. (40)	2015	Biopsy naïve + prior negative biopsy	127	69	5.8	NR	127	3	T2, DWI, DCE, ADC	No	Fusion-TBx (≥1)	TRUS-Bx (10-12)	GS ≥7 and/or MCCL ≥5 mm.
Puech et al. (7)	2013	Biopsy naïve + prior negative biopsy	95	65	10.05	52	95	1.5	T2, DWI, DCE, ADC	No	Fusion-TBx (2)	TRUS-Bx (12)	any 3mm or greater core cancer length or GS ≥ 3 + 4
Sankineni et al. (41)	2015	Biopsy naïve + prior negative biopsy	33	63	8.4	53	33	3	T2, DWI, DCE	Yes	Fusion-TBx (NR)	TRUS-Bx (12)	Gleason > 3 + 4 with 25% biopsy core involvement

**Table 2:** Quality assessment according to QUADAS-2 of the included studies.

STUDY	RISK OF BIAS				APPLICABILITY CONCERNS		
	PATIENT SELECTION	INDEX TEST	REFERENCE STANDARD	FLOW AND TIMING	PATIENT SELECTION	INDEX TEST	REFERENCE STANDARD
Jelid 2017 (18)	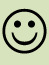	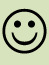	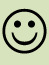	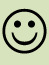	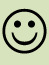	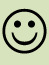	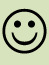
Labra 2020 (19)	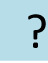	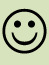	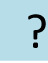	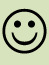	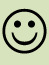	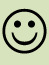	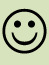
Rastinehad 2014 (20)	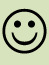	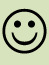	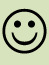	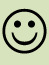	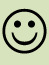	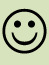	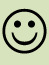
Junker 2015 (21)	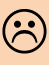	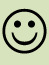	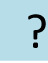	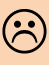	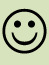	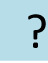	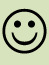
Filson 2016 (23)	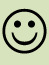	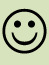	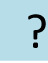	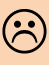	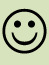	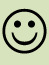	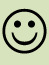
Mariotti 2017 (24)	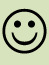	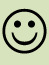	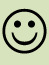	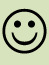	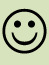	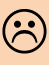	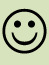
Mariotti 2016 (25)	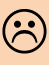	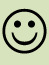	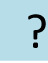	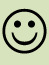	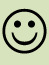	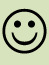	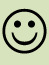
Kongnyuy 2017 (26)	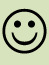	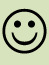	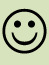	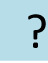	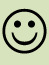	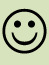	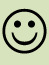
Siddiqui 2015 (27)	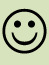	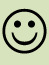	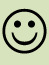	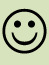	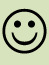	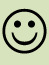	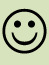
Brock 2015 (28)	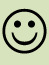	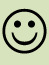	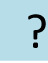	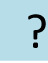	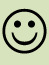	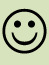	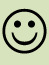
Mozer 2015 (29)	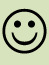	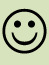	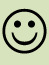	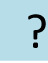	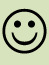	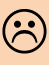	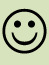
Delongcham 2016 (30)	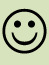	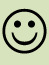	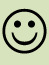	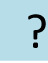	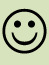	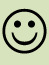	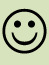
Kaushal 2019 (31)	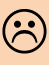	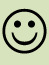	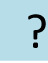	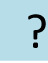	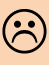	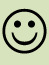	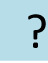
Fujii 2019 (32)	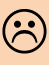	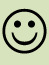	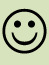	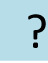	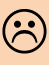	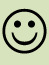	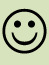
Salami 2015 (33)	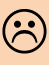	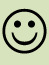	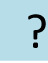	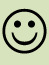	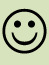	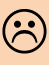	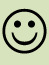
Vourganti 2012 (34)	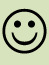	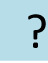	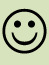	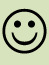	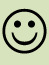	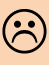	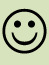
Yarlagadda 2018 (35)	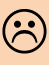	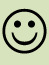	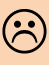	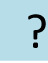	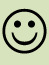	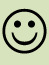	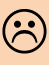
Zhu 2018 (36)	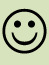	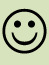	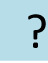	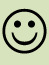	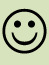	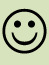	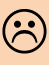
Gorski 2015 (37)	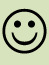	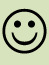	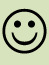	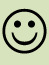	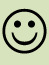	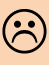	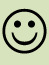
Fiard 2013 (8)	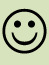	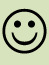	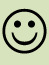	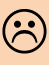	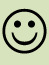	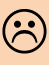	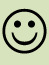
Sonn 2013 (38)	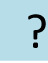	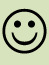	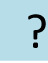	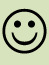	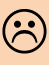	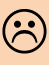	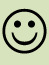
Wysock 2013 (39)	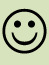	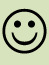	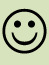	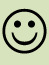	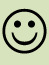	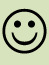	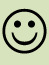
Ukimura 2015 (40)	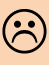	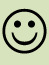	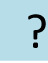	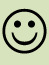	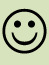	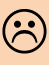	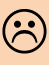
Puech 2013 (7)	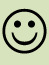	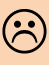	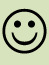	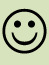	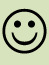	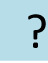	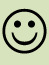
Sankineni 2015 (41)	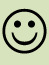	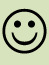	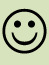	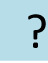	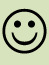	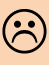	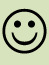

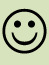
 Low Risk; 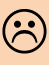 High Risk; 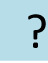 Unclear Risk.

**Table 3:** Quality assessment according to Cochrane Collaboration Risk of Bias Tool.

	Random sequence gene-ration (selection bias)	Allocation concealment (selection bias)	Blinding of participants and researchers (performance bias)	Blinding of outcome assessment (detection bias)	Incomplete outcome data (attrition bias)	Selective reporting (reporting bias)	Other bias
Baco 2016 (22)	Low risk	Low risk	Unclear risk	High risk	Low risk	Low risk	Unclear risk

### 3.3 MRI-TB Compared With SB for Prostate Cancer Detection

#### 3.3.1 Performance of MRI-TB Compared With SB in Clinically Significant and Insignificant PCa Diagnosis

**Figure 3:**
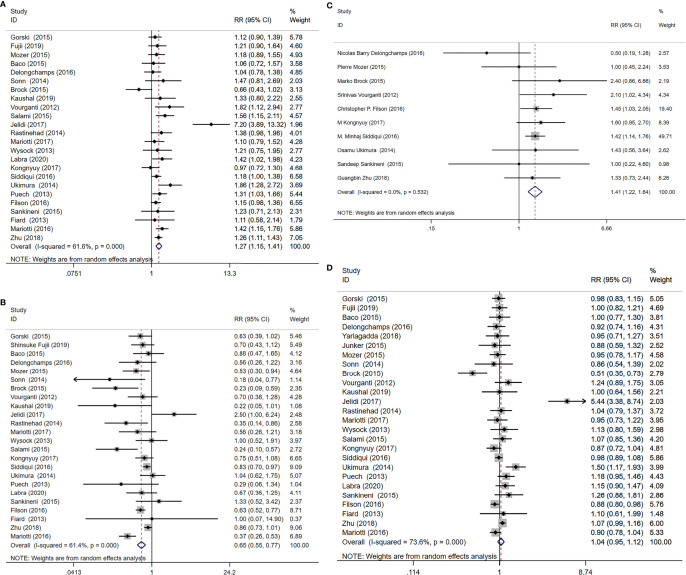
MRI/Transrectal Ultrasound (TRUS) fusion guided-targeted biopsy (MRI-TB) versus transrectal ultrasound-guided biopsy (SB) for the detection of **(A)** clinically significant prostate cancer, **(B)** clinically insignificant prostate cancer, **(C)** high-risk prostate cancer, **(D)** overall prostate cancer.

A total of 24 study cohorts, including 5712 participants were included in the analysis. The rate of diagnosis of clinically significant PCa using MRI-TB was significantly higher compared with the rate for TRUS (RR=1.27, 95% CI =1.15-1.4, p<0.001, I^2^ = 61.6%, **Figure 3A**). MRI-TB diagnosed fewer cases of clinically insignificant PCa compared with TRUS biopsy (RR=0.65, 95% CI=0.55-0.77, p<0.001, I^2^ = 61.4%, **Figure 3B**). Subgroup analysis showed that in the biopsy naïve population, the rate of detection of clinically significant PCa was not significantly different between MRI-TB and SB (RR=1.13, 95% CI =0.99-1.27, p=0.063; I^2^ = 0%). On the other hand, clinically insignificant PCa cases diagnosed through MRI-TB diagnosed were significantly fewer compared with those detected through SB (RR=0.65. 95% CI=0.51-0.84, p=0.001; I^2^ = 0%). In the previous negative biopsy group, no significant differences were reported in clinical significance between the groups (RR=1.29, 95% CI =0.90-1.85, p=0.164, I^2^ = 68.7%). Similarly, MRI-TB showed lower rates of clinically insignificant PCa compared with SB (RR=0.31, 95% CI=0.17-0.58, p<0.001, I^2^ = 48%). In the mixed biopsy population, significant differences were observed in diagnosis of clinically significant PCa (RR=1.36, 95%CI=1.16-1.60, p<0.001, I^2^ = 72.3%) and clinically insignificant PCa using the two methods (RR=0.72, 95% CI=0.57-0.9, p=0.005, I^2^ = 65.4%, **Table 4**).

**Table 4:** Subgroup analyses of MRI-TB versus SB in the diagnosis of prostate Cancer.

Prior biopsy status	Outcome	Number of studies	Model	RR(95% CI)	*P* value	I^2^
biopsy naïve	significant	5	Random	1.13 (0.99,1.27)	*P*=0.063	0%
	insignificant	5	Random	0.65 (0.51-0.84)	*P*=0.001*	0%
	high-risk	2	Random	0.74 (0.38-1.45)	*P*=0.381	16.8%
	overall	6	Random	0.97 (0.89-1.06)	*P*=0.381	0%
Previous negative biopsy	significant	5	Random	1.29 (0.90,1.85)	*P*=0.164	68.7%
	insignificant	5	Random	0.31 (0.17-0.58)	*P*<0.001*	48%
	high-risk	2	Random	2.20 (1.22-3.97)	*P*=0.009*	0%
	overall	6	Random	0.91 (0.70-1.17)	*P*=0.447	66.8%
Mixed biopsy	significant	13	Random	1.36 (1.16-1.60)	*P*<0.001*	72.3%
	insignificant	13	Random	0.72 (0.57-0.90)	*P*=0.005*	65.4%
	high-risk	5	Random	1.44 (1.22-1.70)	*P*<0.001*	0%
	overall	13	Random	1.14 (0.99-1.31)	*P*=0.079	84.1%

CI, confifidence interval; MRI, magnetic resonance imaging; TB, targeted biopsy; SB, systematic biopsy; RR, relative risk.

Mixed = biopsy naïve and Previous negative biopsy, I^2^ is a measure of between-study heterogeneity, *P value < 0.05.

#### 3.3.2 Performance of MRI-TB Compared With SB in High-Risk PCa Diagnosis

Ten studies including 3804 patients reported high-risk PCa, with MRI-TB detecting more cases of high-risk PCa compared with systematic biopsy (RR=1.41, 95% CI=1.22-1.64, p<0.001, I^2^ = 0%, **Figure 3C**).

#### 3.3.3 Performance of MRI-TB Compared With SB in Overall PCa Diagnosis

The included studies involving 26 cohorts, reported overall PCa detection in 5831 cases. Overall PCa cases detected using MRI-TB method were slightly more compared with cases detected through SB method (RR=1.04, 95% CI=0.95-1.12, I^2^ = 73.6%), at 48.6% (2832/5826) vs 47.9% (2793/5831). However, the detection rates for the two groups were not significantly different (p =0.41, **Figure 3D**).

### 3.4 Performance of MRI-TB Integrated With SB Compared With SB Alone in PCa Diagnosis

#### 3.4.1 Performance of MRI-TB+SB vs SB in Diagnosis of Clinically Significant and Insignificant PCa

**Figure 4:**
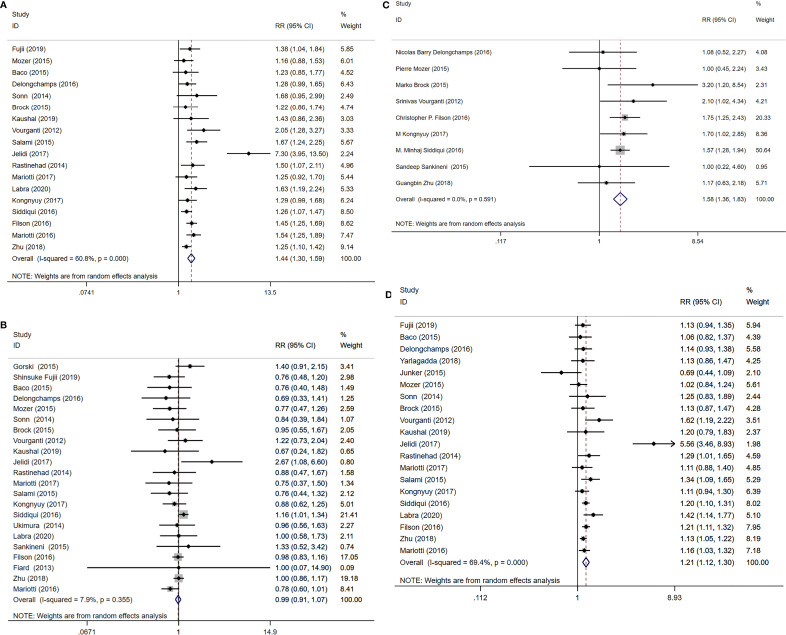
MRI/Transrectal Ultrasound (TRUS) fusion guided-targeted biopsy (MRI-TB) Combined with systematic biopsy(SB) versus SB for the detection of **(A)** clinically significant prostate cancer, **(B)** clinically insignificant prostate cancer, **(C)** high-risk prostate cancer, **(D)** overall prostate cancer.

A total of 18 study cohorts with 5083 participants were included in the analysis. Diagnosis rate of clinically significant PCa using MRI-TB+SB method was significantly higher compared with use of SB alone (RR=1.44, 95% CI=1.30-1.59, p<0.001, I^2^ = 60.8%, **Figure 4A**). However, detection rates of clinically insignificant cases showed no significant differences for MRI-TB+SB and SB methods (RR=0.99, 95% CI=0.91-1.07, p=0.72, I^2^ = 7.9%, **Figure 4B**). Three subgroup analyses based on detection of clinically significant prostate cancer showed statistical differences. Notably, MRI-TB+SB showed a higher detection rate in biopsy naïve cohorts compared with use of SB alone (RR=1.26, 95% CI=1.09-1.46, p=0.002, I^2^ = 0%). Similar results were obtained in individuals with previous negative biopsy (RR=1.56, 95% CI=1.30-1.86. p<0.001, I^2^ = 0%) and mixed biopsy (RR=1.56, 95% CI=1.29-1.89, p<0.001, I^2^ = 78.6%). The two methods did not show significant differences between the three sub-group analysis in detection rates for diagnosis of insignificant PCa (**Table 5**).

**Table 5:** Subgroup analyses of MRI-TB plus SB versus SB in the Diagnosis of prostate cancer.

Prior biopsy status	Outcome	Number of studies	Model	RR(95% CI)	*P* value	I^2^
biopsy naïve	significant	4	Random	1.26 (1.09,1.46)	*P*=0.002*	0%
	insignificant	5	Random	0.88 (0.66-1.18)	*P*=0.395	30.9%
	high-risk	2	Random	0.74 (0.38-1.45)	*P*=0.381	16.8%
	overall	5	Random	1.09 (1.00-1.20)	*P*=0.06	0%
Previous negative biopsy	significant	5	Random	1.56 (1.30,1.86)	*P*<0.001*	0%
	insignificant	5	Random	0.93 (0.70-1.22)	*P*=0.585	0%
	high-risk	2	Random	2.20 (1.22-3.97)	*P*=0.009*	0%
	overall	6	Random	1.21 (1.00-1.46)	*P*=0.048*	50.7%
Mixed biopsy	significant	8	Random	1.56 (1.29-1.89)	*P*<0.001*	78.6%
	insignificant	11	Random	0.99 (0.87-1.13)	*P*=0.910	27%
	high-risk	4	Random	1.62 (1.37-1.91)	*P*<0.001*	0%
	overall	8	Random	1.32 (1.15-1.52)	*P*<0.001*	84.6%

CI, confifidence interval; MRI, magnetic resonance imaging; TB, targeted biopsy; SB, systematic biopsy; RR, relative risk; Mixed, biopsy naïve and Previous negative biopsy; I^2^ is a measure of between-study heterogeneity, *P value < 0.05.

#### 3.4.2 Performance of MRI-TB+SB Compared With SB in High-Risk PCa Detection

Nine studies including 3677 cases reported high-risk PCa detection rates. Detection rates of high-risk PCa cases using MRI-TB+SB were significantlyu higher compared with use of SB alone (RR=1.58, 95% CI=1.36-1.83, p<0.001, I^2^ = 0%, **Figure 4C**).

#### 3.4.3 MRI-TB+SB vs SB in Overall PCa Detection

A total of 20 studies that reported 5220 PCa cases were included in this meta-analysis. MRI-TB+SB exhibited significantly higher overall PCa detection rate compared with SB alone (RR=1.21, 95% CI=1.12-1.30, p<0.001, I^2^ = 69.4%, **Figure 4D**). Subgroup analysis did not show statistically significant differences in detection of biopsy naïve patients using the two methods (p=0.06). However, in previous negative biopsy group (RR=1.21, 95% CI=1.00-1.46, p=0.048, I^2^ = 50.7%) and mixed biopsy patients (RR=1.32, 95% CI=1.15-1.52, p<0.001, I^2^ = 84.6%), the detection rate of MRI-TB+SB for overall PCa cases was significantly higher compared with use of SB alone (**Table 5**).

### 3.5 Sensitivity Analysis

**Figure 5:**
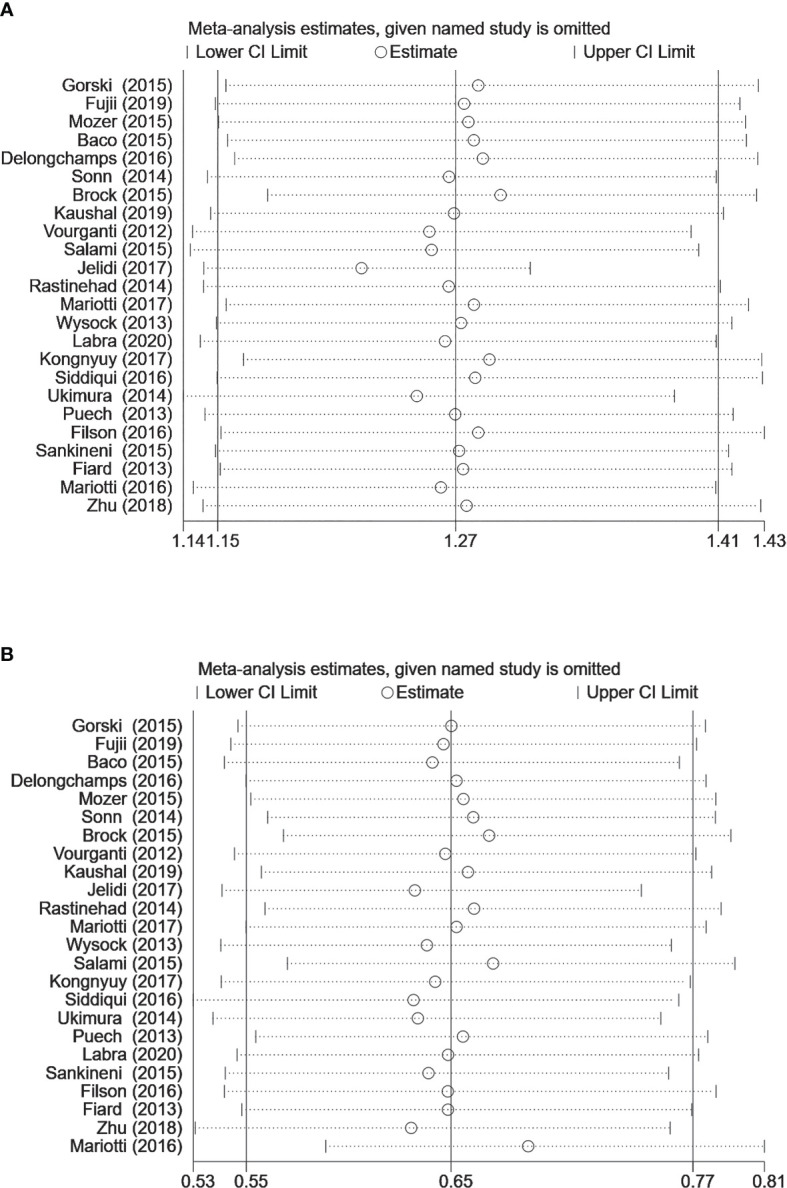
Sensitivity analysis for **(A)** clinically significant prostate cancer detection **(B)** clinically insignificant prostate cancer detection (MRI-TB vs. systematic biopsy). The circles represent the RR estimate and the horizontal lines represent the 95% CI. CI, confidence interval; RR, relative risk.

In the analysis of MRI-TB vs SB to detect prostate cancer, heterogeneity was observed in diagnosis of clinically significant PCa and insignificant PCa. Subgroup analysis showed high heterogeneity in previous negative biopsy and mixed biopsy subgroups and low heterogeneity in the biopsy naïve subgroup, implying that prior biopsy status may not be the source of heterogeneity. Therefore, we performed a sensitivity analysis for clinically significant PCa, and insignificant PCa. Sensitivity analysis did not show any effect on the results when we excluded the studies one by one (**Figure 5**), implying that heterogeneity was caused by differences in other variables. Although subgroup analyses were performed, several variables were not available for correlation analysis.

### 3.6 Publication Bias

**Figure 6:**
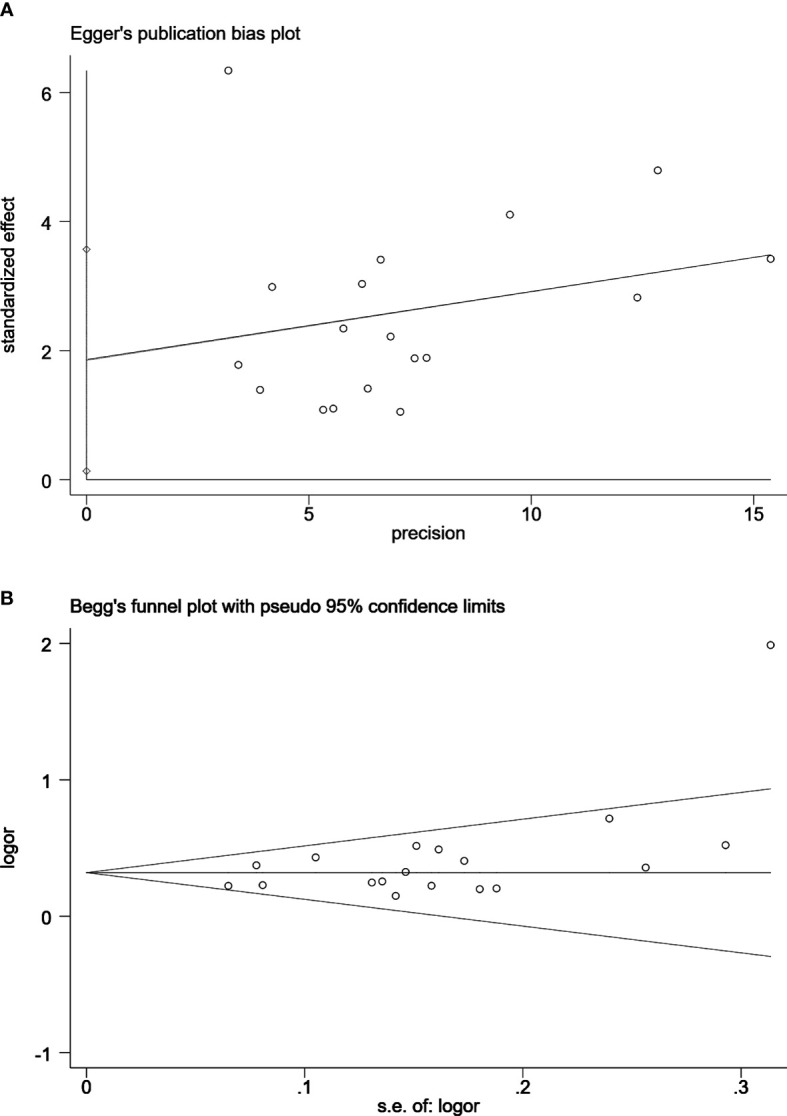
Egger’s publication bias plot **(A)** and Begg’s funnel plot **(B)** for the assessment of publication bias for clinically significant prostate cancer detection by MRI/Transrectal Ultrasound (TRUS) fusion guided-targeted biopsy (MRI-TB) Combined with systematic biopsy(SB) versus SB. Egger’s test, *P*=0.036. Begg’s test, *P*=0.081.

In MRI-TB vs SB analysis of PCa detection, no publication bias was observed using Begg’s test or Egger’s test. In MRI-TB+SB vs SB analysis of PCa detection no publication bias was detected except for clinically significant PCa cases (Egger’s test, P=0.036. Begg’s test, P=0.081) (**Figure 6**).

## 4 Discussion

Although TRUS-guided prostate biopsy is widely used for diagnosis of prostate cancer, it is not effective in determining tumor location (17). Furthermore, incidence of clinically relevant tumor false-negative biopsy using TRUS is approximately 47%, which can delay treatment of tumors with high Gleason scores (42). Multiparametric MRI comprises contrast-enhanced dynamic imaging, T2-weighted, and diffusion-weighted strategies, and is considered the most sensitive imaging approach for PCa detection (11). Prostate gland mp-MRI is a valuable imaging technique for patients that require targeted prostate biopsy as it effectively identifies higher grades and PCa volume compared with systematic 12-core prostate biopsy. Therefore, mp-MRI increases accuracy of prostate biopsy analysis by guiding clinicians to suspicious areas during biopsy rather than random sampling (19). The findings of this systematic review show that MRI-TRUS fusion biopsy diagnoses more clinically significant PCa and high-risk PCa compared with systematic biopsy, with lower rate of detection of clinically insignificant PCa, thus avoiding consequent overtreatment.

Analysis showed that MRI-TB has a higher detection rate of clinically significant PCa and high-risk PCa compared with traditional TRUS-guided biopsy. On the other hand, the rate of detection of clinically insignificant PCa using MRI-TB was lower compared with the rate of detection using traditional TRUS-guided biopsy. MRI/TRUS fusion biopsy did not show significant differences in overall PCa diagnosis compared with systematic biopsy. These results are consistent with previous findings that MRI-TB diagnoses more clinically significant PCa and fewer clinically insignificant PCa cases compared with standard biopsy (12, 13). Similarly, in the study carried out by Pepe et al., they included a total of 1032 patients suspicion of cancer and found that MRI targeted fusion biopsy had a lower detection rate of clinically insignificant prostate cancer than transperineal saturation biopsy (43). However, the previous systematic reviews did not explore the detection rate of MRI-TB for high-risk PCa. Currently, very few systematic reviews and meta-analyses have explored detection rate of high-risk PCa using MRI-TB. Similar findings were reported by Tang et al. on high-risk PCa detection, however, meta-analysis mainly evaluated MRI/TRUS fusion 3D model-guided targeted biopsy for PCa (44). Patients with high-risk prostate cancer are at an increased risk of biochemical recurrence, metastatic progression and cancer-related death following primary treatment compared with patients with low-risk or intermediate-risk disease (45). Individuals with high-risk PCa have high risk of systemic or local relapse and are more likely to experience symptoms and/or higher risk of death (17). Therefore, we explored high-risk PCa detection in the present meta-analysis. Our findings show that MRI-TB may help clinicians to effectively screen high-risk PCa, thus improving treatment decisions, which are consistent with previous findings (27).

Analysis of the 3 PCa diagnosis methods (including MRI-TB combined with SB) showed that MRI-TB+SB was superior in detection of overall, clinically significant, and high-risk PCa, which was consistent with reports from previous studies (23, 27–30). Rapisarda et al. compared the coincidence rate in the detection of clinically significant PCa cancers (GS≥7) between combined biopsy (fusion plus standard) and final histological report to 73.6%. They suggest that targeted biopsy combined with systematic biopsy can further improve the detection rate of clinically significant prostate cancer and reduce the missed diagnosis rate with targeted biopsy alone (46). This observation coincides with our findings.However, MRI-TB+SB did not exhibit significantly different diagnostic rate for clinically insignificant PCa compared with SB method. Therefore, MRI-TB+SB did not reduce detection of clinically insignificant PCa compared with SB.

This review had a few limitations. Firstly, most of the included studies had paired designs, therefore, the analysis introduces bias because conclusions from such data are limited to MRI patients with suspicious findings using MRI-TB, and systematic biopsy. Secondly, MRI image quality and definitions of clinically significant and high-risk PCa were different across centers, causing heterogeneity. Finally, integrating MRI-TB with SB improves rate of detection of overall clinically significant cases, and high-risk PCa case compared with other biopsy methods. However, MRI-TB+SB needs more biopsy cores. Therefore, further studies should be performed to determine MRI-TB+SB results in more complications compared with systematic biopsy.

Finally, we note some new directions in PCa research and give an outlook to the future research. Recently, radiomics and genomics have increasingly become a prospective topic in the field of prostate cancer research. A combination of these fields, radiogenomics, is an emerging fifield that studies the correlation between image phenotypes and genomics inside a tumor. Prostate cancer has been extensively investigated using radiogenomics, with experiments between quantitative image features and single gene expression, which have yielded promising results (47). Some studies showed that radiomic features correlation with Gleason score and PIRADS sum scores (48–50). In the detection of clinically significant PCa, Combining radiological and clinical radiomic models was indeed effective in predicting clinically significant PCa in patients with a PIRADS score of three or more. It is possible to further improve the radiomic potential of this issue by developing different models automatically, using machine learning and artificial intelligence techniques, and by creating nomograms (51). However, At present, radiogenomics holds great promise, but it is a new area of research, the use of radiogenomics in clinical practice regarding prostate cancer does not have any utility or effectiveness.We need to fill many gaps to achieve clinical implementation of radiogenomics.

## 5 Conclusion

The findings of this study show that MRI-TB has higher detection rate for clinically significant and high-risk PCa cases, and fewer rate for clinically insignificant PCa cases compared with systematic protocols. Combination of MRI-TB with SB improves PCa detection in compared with use of either of the methods alone. However, MRI-TB+SB does not reduce the diagnosis rate of clinically insignificant PCa. The findings of this study provide information for clinicians and patients on the risks and benefits of using MRI-TB or systematic biopsy.

## Data Availability Statement

The original contributions presented in the study are included in the article/supplementary material. Further inquiries can be directed to the corresponding authors.

## Author Contributions

JX and CJ have contributed equally. JX, conception, manuscript preparation, data collection and analysis. CJ, conception, data analysis, manuscript editing and manuscript review. ML, Study design, manuscript editing and manuscript review. KS, study design and manuscript review. ZJ, data collection and analysis. ZD and XG, substantial contributions, manuscript editing and manuscript review. All authors contributed to the article and approved the submitted version.

## Funding

This study was supported by grants from Shenzhen Science and Technology Innovation Committee (JCYJ20170413161913429), Shenzhen Key Medical Discipline Construction Fund (SZXK052) and Sanming Project of Medicine in Shenzhen (SZSM201612027).

## Conflict of Interest

The authors declare that the research was conducted in the absence of any commercial or financial relationships that could be construed as a potential conflict of interest.

## Publisher’s Note

All claims expressed in this article are solely those of the authors and do not necessarily represent those of their affiliated organizations, or those of the publisher, the editors and the reviewers. Any product that may be evaluated in this article, or claim that may be made by its manufacturer, is not guaranteed or endorsed by the publisher.
